# Supplementary material for: A whole-animal phenotypic drug screen identifies suppressors of atherogenic lipoproteins
Source: eLife. 2026 Aug 27;14:RP105314. doi: 10.7554/eLife.105314 (PMC13521566; doi:10.7554/eLife.105314)
Supplement: Supplementary file 2 [file elife-105314-code1.zip › JHDL_primary_result/Ferron (8-Hydroxy-7-iodo-5-quinolinesulfonic acid)/Ferron (8-Hydroxy-7-iodo-5-quinolinesulfonic acid)_JHDL_29_009_039_2019_12_06_12_17.pdf]

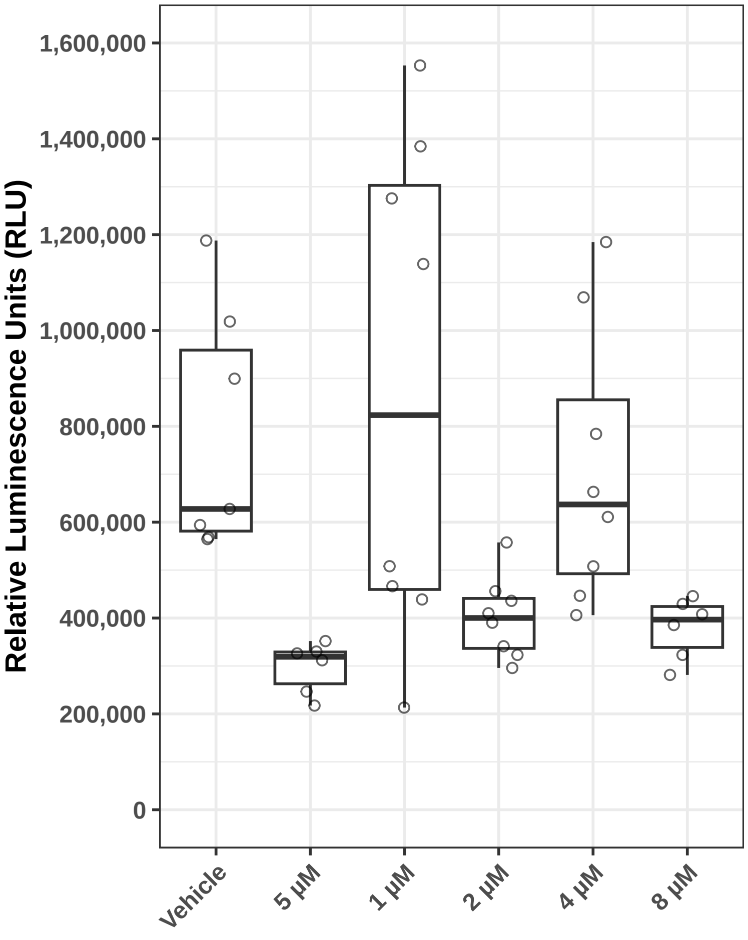
Relative Luminescence Units (RLU)
1,600,000
1,400,000
1,200,000
1,000,000
800,000
600,000
400,000
200,000
0
Vehicle
5 μM
1 μM
2 μM
4 μM
8 μM
